# Supplementary material for: The causal role of circulating inflammatory markers in osteoporosis: a bidirectional Mendelian randomized study
Source: Front Immunol. 2024 Jul 18;15:1412298. doi: 10.3389/fimmu.2024.1412298 (PMC11291241; doi:10.3389/fimmu.2024.1412298)
Supplement: Supplementary file 1 [file DataSheet_1.zip › supplementary material/OSTEOPOROSIS-CIM/GCST90274793.tsv.gz_leave_one_out.pdf]

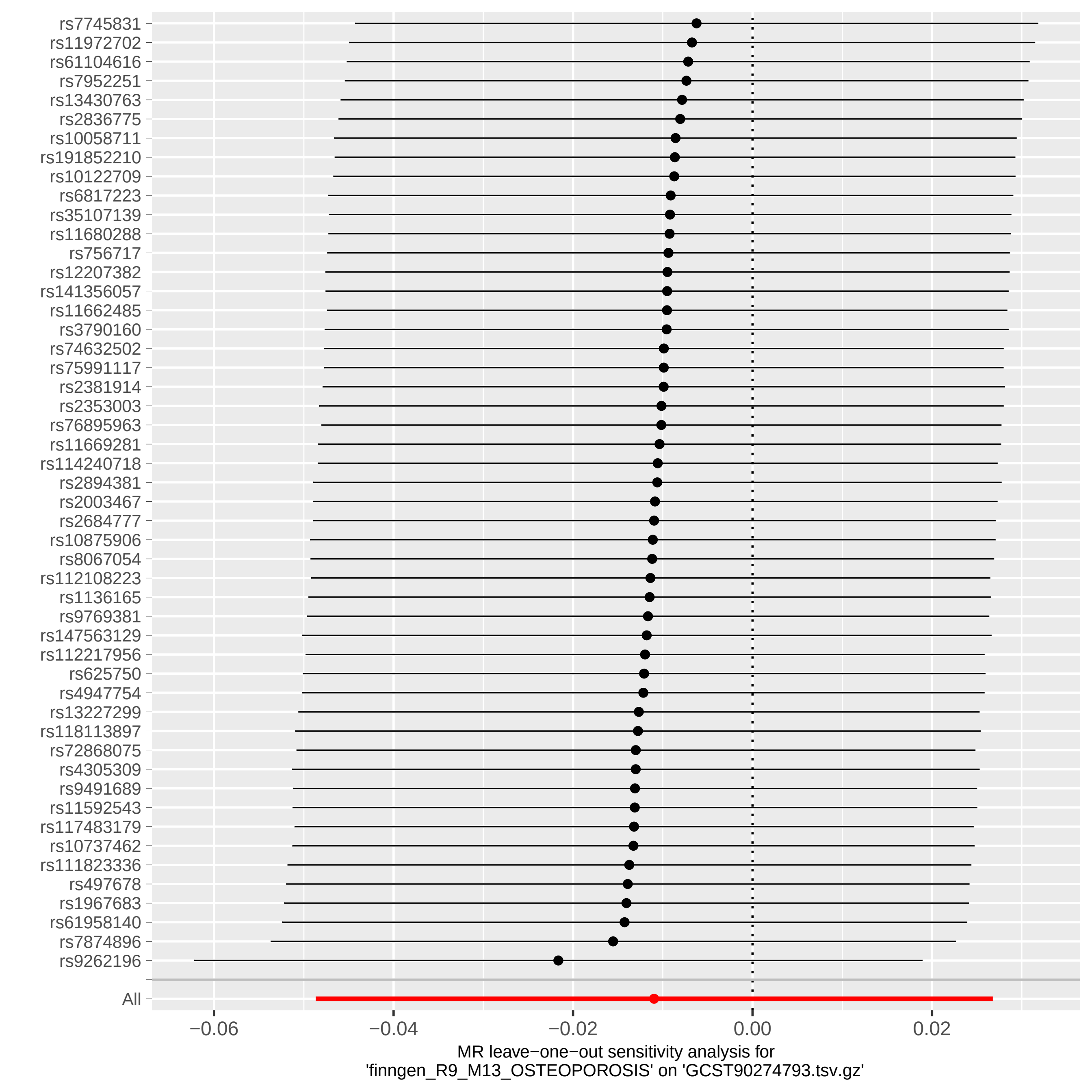
rs7745831
rs11972702
rs61104616
rs7952251
rs13430763
rs2836775
rs10058711
rs191852210
rs10122709
rs6817223
rs35107139
rs11680288
rs756717
rs12207382
rs141356057
rs11662485
rs3790160
rs74632502
rs75991117
rs2381914
rs2353003
rs76895963
rs11669281
rs114240718
rs2894381
rs2003467
rs2684777
rs10875906
rs8067054
rs112108223
rs1136165
rs9769381
rs147563129
rs112217956
rs625750
rs4947754
rs13227299
rs118113897
rs72868075
rs4305309
rs9491689
rs11592543
rs117483179
rs10737462
rs111823336
rs497678
rs1967683
rs61958140
rs7874896
rs9262196
All
−0.06
−0.04
−0.02
0.00
0.02
MR leave−one−out sensitivity analysis for
'finngen_R9_M13_OSTEOPOROSIS' on 'GCST90274793.tsv.gz'
